# Traditional application and modern pharmacological research of *Eucommia ulmoides *Oliv.

**DOI:** 10.1186/s13020-021-00482-7

**Published:** 2021-08-06

**Authors:** Lichuang Huang, Qiang Lyu, Wanying Zheng, Qiao Yang, Gang Cao

**Affiliations:** School of Pharmacy, Zhejiang Chinese Medical University, 548 Binwen Road, Hangzhou, 310053 China

## Abstract

As a Traditional Chinese Medicine, *Eucommia ulmoides Oliv.* has been used for the treatment of various diseases since ancient times, involving lumbar pain, knee pain, osteoporosis, hepatoprotection, paralysis, intestinal haemorrhoids, vaginal bleeding, abortion, spermatorrhoea, foot fungus, anti-aging etc. With the developing discovery of *E. ulmoides* extracts and its active components in various pharmacological activities, *E. ulmoides* has gained more and more attention. Up to now, *E. ulmoides* has been revealed to show remarkable therapeutic effects on hypertension, hyperglycemia, diabetes, obesity, osteoporosis, Parkinson’s disease, Alzheimer’s disease, sexual dysfunction. *E. ulmoides* has also been reported to possess antioxidant, anti-inflammatory, neuroprotective, anti-fatigue, anti-aging, anti-cancer and immunoregulation activities etc. Along these lines, this review summarizes the traditional application and modern pharmacological research of *E. ulmoides*, providing novel insights of *E. ulmoides* in the treatment of various diseases.

## Introduction

*Eucommia ulmoides Oliver*, a plant belonging to the Eucommiaceae family, once widespread in the Northern Hemisphere, disappeared from other parts of Europe and North America due to the Quaternary Ice Age, and survives only in China [1]. As the utility and value of *E. ulmoides* has been recognized, the planting of *E. ulmoides* has been expanded. Hitherto the main production areas include the central and northern subtropical and southern temperate zones, and it has been successfully introduced to the United States, Britain, France, Hungary, Russia, Japan, Korea and other countries. *E. ulmoides* was first recorded in the Shen Nong Ben Cao Jing and has been used for over 2,000 years. The bark, leaves, seed, and the even male flowers are commonly utilized as medical remedies and performed for investigation of pharmacy. As a particularly well-known traditional Chinese medicine, Chinese Pharmacopoeia documents the main functions of *E. ulmoides* bark are to nourish the liver and kidneys, strengthen the tendons and bones, and calm the fetus; *E. ulmoides* leaves are to nourish the liver and kidneys, strengthen the tendons and bones, which are similar to the bark and sometimes used as an alternative medicine.

Modern pharmacological studies reveal the remarkable therapeutic effects of *E. ulmoides* in hypertension, hyperglycemia, diabetes, obesity, osteoporosis, Parkinson’s disease, Alzheimer’s disease, aging, and sexual dysfunction. The iridoids and lignans are the major active constituents of *E. ulmoides*. Aucubin and geniposide isolated from *E. ulmoides*, both belonging to iridoids, have been extensively and profoundly studied for their biological activities involving anti-hypertension, anti-diabetes, neuroprotection, anti-cancer, anti-inflammatory, anti-osteoporotic, hepatoprotection and kidney protection etc. [2, 3], and these biological activities are possessed by the extract of *E. ulmoides* bark or leaves as well. Overall, the extensive biological activities made *E. ulmoides* a promising therapeutic to be widely used in clinical therapy. The aim of this review was to provide a comprehensive overview on the traditional application and the modern pharmacological research correlative with *E. ulmoides*, providing novel insights of *E. ulmoides* in the treatment of numerous diseases.

## Traditional application of *E. ulmoides*

**Fig. 1:**
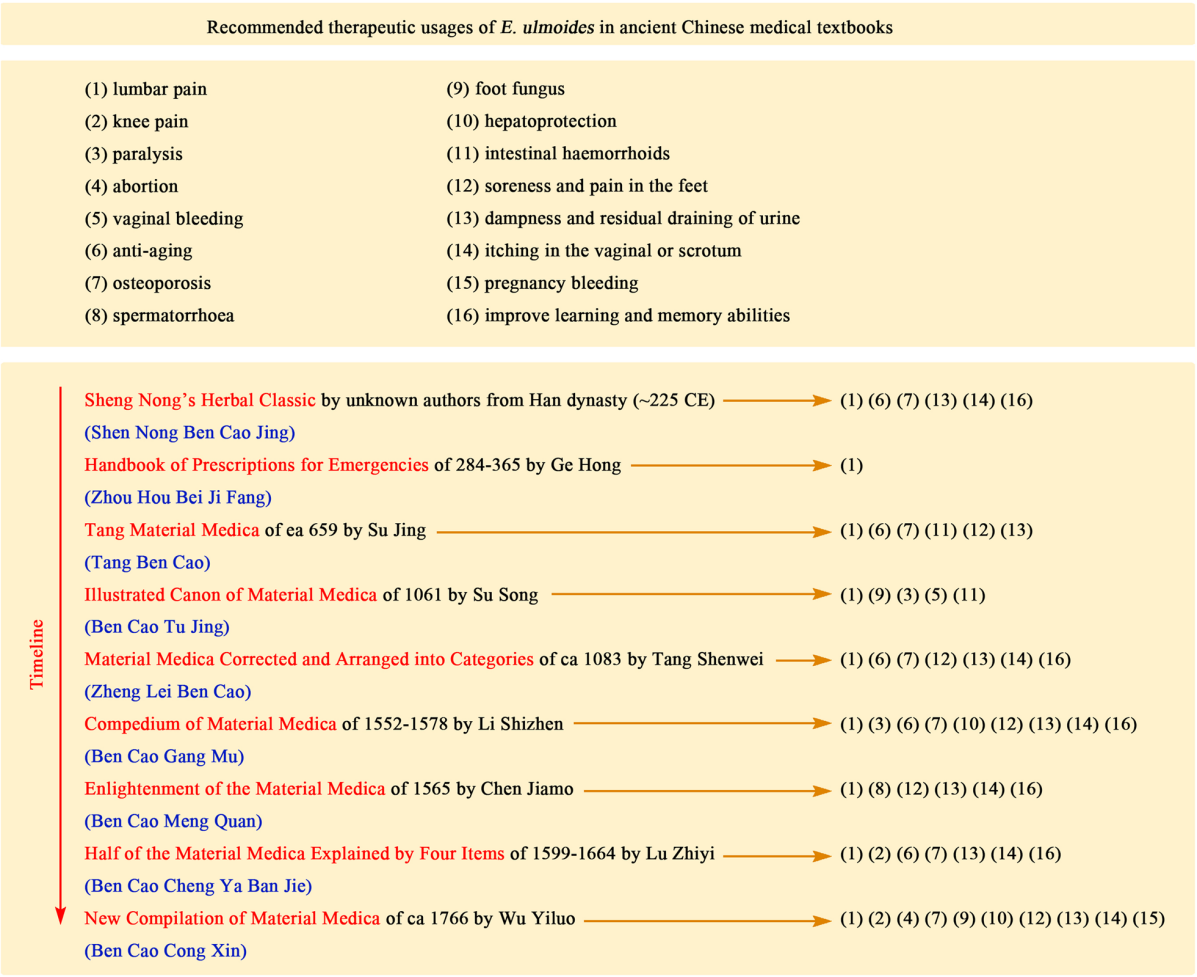
Traditional applications of *E. ulmoides* recorded in ancient Chinese medical textbooks

*Eucommia ulmoides *was first recorded in “Sheng Nong’s Herbal Classic (Shen Nong Ben Cao Jing)” of the Han Dynasty in China, which deals with the alias, origin, properties, flavour and functions of *E. ulmoides*. Nowadays, *E. ulmoides* has been officially recognized as a medicinal plant and listed in Chinese Pharmacopeia. As recorded in ancient medical textbooks, *E. ulmoides* was recommended for the treatment of lumbar pain, knee pain, osteoporosis, improve learning and memory abilities, hepatoprotection, paralysis, intestinal haemorrhoids, vaginal bleeding, itching in the vaginal or scrotum, dampness and residual draining of urine, abortion, pregnancy bleeding, spermatorrhoea, soreness and pain in the feet, foot fungus, anti-aging (Fig. 1).

## Biological activities of *E. ulmoides*

### Cardiovascular system regulation activities of *E. ulmoides*

#### Anti-hypertensive activities

Since the 1970s, the main pharmacological active compound, which termed (+)-pinoresinol di-β-d-glucoside (PG), for the anti-hypertensive activities of *E. ulmoides* have been investigated and reported [4]. In a subsequent clinical trial study reported in 1983, 18 men and 35 women with mild hypertension significantly decreased their blood pressure after a course of treatment with aqueous extract of *E. ulmoides* leaves, led to the potential of *E. ulmoides* as a promising candidate for the treatment of hypertensive diseases being identified [5].

A review conducted by He et al. outlined the research results of its pharmacological mechanism until 2014, that is, *E. ulmoides* exerts its function via inhibiting cAMP activity and Ca^2+^ internal flow (Analysis based on several studies from the 1970s to the 1990s), regulating NO and the renin-angiotensin system, relaxing blood vessels, as well as increasing coronary flow [6]. However, these summaries of *E. ulmoides* anti-hypertensive mechanism based on individual components instead of integral component tails are not absolute correct. A study conducted in 2010 revealed that *E. ulmoides* leaf lignans rather than iridoids capable of exerting antihypertensive activities *in vivo*. The lignans (150 and 300mg/kg) showed a dose-dependent hypotensive effect in spontaneously hypertensive rats (SHRs), and decreased plasma renin activity and Ang II concentration. The lignans at 300mg/kg dramatically increased plasma NO levels. Ex vitro trails showed the lignans (0.05, 0.2, 0.4 and 0.8 mg/ml) relaxed mesenteric arteries in a dose-dependent manner. However, iridoids from *E. ulmoides* leaf (15, 30 and 60mg/kg by intravenous administration) showed no effect on blood pressure. Moreover, combination of lignans (300 mg/kg) and Iridoids (125 mg/kg) by intragastric administration showed no synergistic effect [7]. Hence, although the earlier studies summarized by the review proposed that the compounds of iridoids, that is, asperuloside, geniposidic acid and genipin, exhibited varying degrees of anti-hypertensive effects in *in vivo* or *in vitro* trials, total iridoids from *E. ulmoides* leaf does not possess anti-hypertensive activity [6]. As we all know, phytomedicines consist of a complex range of compounds, and even in cases where the same fractions are isolated for *in vivo* or *in vitro* studies, the strength of their pharmacological activities are balanced by the counteracting pharmacological effects of the distinct compounds, especially in the case of *in vivo* assays, which involving complex biological regulatory mechanisms inherent in the organism.

In addition to *E. ulmoides* leaf, the lignans isolated from *E. ulmoides* bark showed similar anti-hypertensive activities *in vivo* [8–10]. Very recently, the anti-hypertensive effects of *E. ulmoides* male flowers were well documented. The aqueous extract of *E. ulmoides* male flowers (0.05, 0.10 and 0.20g/kg) dose-dependently reduced the blood pressure, and its effect via promoting expression of angiotensin-converting enzyme 2 (ACE2), of which function as a carboxypeptidase to catalyze the conversion of Ang II into Ang-(1–7). Subsequent trails were performed with ACE2 inhibitor DX600 and Ang-(1–7)-Mas receptor antagonist A-779, and the results showed the therapeutic effects of *E. ulmoides* male flowers in SHRs were reversed [11]. This study indicated that the anti-hypertensive activities of *E. ulmoides* may via the activation of ACE2-Ang-(1–7)-Mas signalling pathways, despite more investigations should be performed to establish whether this pathway is existing in *E. ulmoides* leaf or bark treatment.

Taken together, *E. ulmoides* has been identified as possessing anti-hypertensive activities for over forty years. Numerous individual components of the lignans and iridoids from *E. ulmoides*, involving PG, liriodendrin, (+)-syringaresinol, syringin, asperuloside, geniposidic acid and genipin, have been revealed to be able to lower blood pressure through inhibiting cAMP activity, Ca^2+^ internal flow, regulating NO and the renin-angiotensin system, relaxing blood vessels, and increasing coronary flow [6]. Intriguingly, the active constituent of *E. ulmoides* lignans showed anti-hypertensive activities in *in vivo* experiments, whereas the iridoids did not. Whether the opposite tendencies of action of the different components in the iridoids counteract the anti-hypertensive activities remains to be further investigated. Of note, the hypotensive effect of *E. ulmoides* lignans is not only exist in the pathological state of hypertension, but also shows hypotensive activities even in the normal physiological state in a dose-dependent manner [7]. Additionally, in parallel to the leaves and bark of *E. ulmoides*, its male flowers also exhibit anti-hypertensive activities. Future studies need to focus on whether interference with the ACE2-Ang-(1–7)-Mas signalling pathway is present in both *E. ulmoides* leaves and bark in the therapeutic mechanism of hypertension.

#### Other cardiovascular system regulation activities

In the last decade, incidentally, in studies of the anti-hypertensive activities of *E. ulmoides*, it was discovered that lignans isolated from the bark were able to antagonize the expression of aldose reductase (AR) (300 mg/kg into SHRs), similarly to epalrestat (100 mg/kg), and thus exhibiting effects of against hypertensive vascular remodeling or against hypertensive renal injury [8–10]. AR is a multifunctional enzyme that reduces aldehydes. The correlation between AR and cardiovascular diseases particularly in the diabetic context has been summarized in an excellent review in 2010. Overall, AR is implicated in excess smooth muscle cell (SMC) growth, and its inhibition manifested as an amelioration of diabetes cardiac diseases and ischemia/reperfusion (I/R)-injured hearts [12]. Although there is still a deficiency of critical evidence that *E. ulmoides* lignans can act as an AR antagonist to improve cardiovascular disease, it is still a promising candidate for the treatment of diabetic heart disease or I/R-injured hearts.

Comparing to *E. ulmoides* extracts or active fraction groups, the individual active components of *E. ulmoides* have been more intensively studied in the treatment of cardiac diseases, involving cardiac hypertrophy, cardiac fibrosis and cardiac remodelling etc., and their associated mechanisms over the past year [13–21]. For instance, both *in vivo* and *in vitro*, aucubin has been found protecting against pressure overload-induced cardiac remodelling via β3-AR-nNOS cascades and it has been found protecting against myocardial infarction-induced cardiac remodeling via nNOS/NO-regulated oxidative stress [19, 21]. Meanwhile, geniposide has been revealed protecting against obesity-related cardiac injury through AMPKα- and sirtuin (Sirt1)-dependent mechanisms, etc. [16]. However, these effects have not yet been reported for *E. ulmoides* extracts or active fraction groups and needed further investigation to corroborate.

### Anti-osteoporotic activities of *E. ulmoides*

*Eucommia ulmoides *has been unveiled to possess therapeutic effects on hypoestrogenemia after menopause result in osteoporosis, disuse-induced osteoporosis, senescence caused osteoporosis and lead affect bone composition and mineralization result in osteoporosis, and these therapeutic effects on osteoporotic models have been verified in ovariectomy (OVX) rats, hind limb suspension (HLS) rats, senescence-accelerated mice and lead acetate-induced bone loss rats. Mechanistic studies have revealed its anti-osteoporotic activities were referring to the modulation of the OPG/RANKL system in osteoblastic cells [22–27].

As early as 1998, a study reported the collagen synthesis promoting effect of *E. ulmoides* [28]. This biological activities of promoting collagen synthesis are favor for bone strengthening as modern studies have conclusively proven that a wide variety of genes encoding proteins involved in collagen synthesis, structure, processing, post-translational modification etc. have been showed to cause osteogenesis imperfect [29]. The above study also found that after fractionation of the methanolic extract of *E. ulmoides* leaves with n-hexane, AcOEt and Acetone sequentially, only methanol extract and Acetone fraction showed a dose-dependent pro-collagen synthesis effects in *in vivo* experiments in false aged model rats. Further *in vivo* trails have also shown that geniposidic acid and aucubin are the active components in the pro-collagen synthesis of *E. ulmoides* [28]. Similarly, a study reported in 2003 also adopted a fractionation approach with a view to screening *E. ulmoides* for bone-strengthening active components. Of note, methanol extract of *E. ulmoides* cortex was fractionated with n-hexane, chloroform, ethylacetate, butanol sequentially and performed with several *in vitro* trails. Details of the two studies in 1998 and 2003 are collated in Table 1[30]. As it showed, in study of Ha et al., each fractions or components participate in activities of osteoblast or osteoclast: (1) butanol fraction, aqueous fraction as well as geniposide and aucubin promoting proliferation and differentiation on osteoblast, (2) chloroform fraction and aucubin relating to the maturation of osteoblast, (3) chloroform, AcOEt fraction and geniposide show the effects on the synthesis of collagen participating in the matrix formation of osteoblast, (4) both chloroform and AcOEt fraction showed potent induction of growth hormone (GH) release, through which approximately regulating bone maturation and remodeling, and (5) geniposidic acid and aucubin exhibited inhibition on osteoclast in dose-dependent manner. In line with anticipation, these effects on activities of osteoblast and GH release were present in Methanol extract. Collectively, these analyses suggest that the overall components of *E. ulmoides* cortex (extract) may be more effective in preventing and treating osteoporosis than the individual components.

**Table 1:** Anti-osteoporosis activities of *E. ulmoides*

Compounds	Subjects	Dose	Effects	Notes	References
Methanol extract of *E. ulmoides* leaf (MEEuL)	False aged model rats	1.8 and 2.7 g of dried *E. ulmoides* leaf/kg	MEEuL promoted collagen synthesis in a dose-dependent manner (increase of 68% and 131% respectively)	These fractions were fractioned with organic reagent sequentially	[28]
N-hexane fraction of the methanol extract of leaf (NF-MEEuL)		1.5 and 3.1 g of dried *E. ulmoides* leaf/kg	No significant effect on collagen synthesis		
AcOEt fraction of the methanol extract of leaf (AEF-MEEuL)		1.5 and 2.9 g of dried *E. ulmoides* leaf/kg	No significant effect on collagen synthesis		
Acetone fraction of the methanol extract of leaf (AF-MEEuL)		1.5 and 3.0 g of dried *E. ulmoides* leaf/kg	AF-MEEuL promoted collagen synthesis in a dose-dependent manner (increase of 57% at dose of 3.0 g of dried *E. ulmoides* leaf/kg)		
Methonal fraction of the methanol extract of leaf (MF-MEEuL)		1.5 and 3.0 g of dried *E. ulmoides* leaf/kg	No significant effect on collagen synthesis		
Geniposidic acid		25, 50 and 100 mg/kg	Geniposidic acid promoted collagen synthesis in a dose-dependent manner (increase of 79%, 82%, 95% respectively)		
Aucubin		25 and 50 mg/kg	Aucubin promoted collagen synthesis in a dose-dependent manner (increase of 99% at dose of 50 mg/kg)		
Methanol extract of *E. ulmoides* cortex (MEEuC)	Osteoblast, MG- 63 and Saos-2 (osteoblast-like cells); mouse bone marrow cells and ST-2 cells (osteoclast-like cells)	1 mg/ml in GH inducing test; 1 × 10 ^−1^–1 × 10 ^−11^ mg/ml in MTT test and ALP activity assay; 1 × 10 ^−1^–1 × 10 ^−7^ mg/ml in nhibition of osteoclast	MEEuC showed potent induction of growth hormone (GH) release in the same extent as GH releasing factors (GRF) 1.0 mMMEEuC promoted cell proliferation and differentiation of osteoblast 32% at 1 × 10 ^−8^ mg/ml MEEuC promoted maturation of osteoblast 10% at 1 × 10 ^−1^ mg/ml. MEEuC inhibited around 35% of osteoclast at 1 × 10 ^−3^–1 × 10 ^−5^ mg/ml	MTT assay shows the proliferation and differentiation of osteoblast using osteoblast-like cells. ALP activity detection assays the activity of ALP, a specific enzyme relating to the maturation step of osteoblast Praline incorporation test shows the effects on the synthesis of collagen participating in the matrix formation of osteoblast	[30]
N-hexane fraction of the methanol extract of cortex (NF-MEEuC)		Each fractions: 20 µg/ml in GH inducing test; 1 × 10 ^−1^–1 × 10 ^−11^ mg/ml in MTT test and ALP activity assay; 1 × 10 ^−2^–1 × 10 ^−6^ mg/ml in Praline incorporation test	NF-MEEuC showed potent induction of GH release in the same extent as GRF 0.5 mM		
Chloroform fraction of the methanol extract of cortex (CF-MEEuC)			CF-MEEuC showed potent induction of GH release in the same extent as GRF 0.5 mM CF-MEEuC promoted maturation of osteoblast 38% at 1 × 10 ^−6^ mg/ml CF-MEEuC promoted synthesis of collagen participating in the matrix formation of osteoblast 40% at 1 × 10 ^−3^ mg/ml		
Ethylacetate fraction of the methanol extract of cortex (EF-MEE)			EF-MEE showed potent induction of GH release in the same extent as GRF 0.5 mM EF-MEE promoted synthesis of collagen participating in the matrix formation of osteoblast 20% at 1 × 10 ^−5^ mg/ml		
Butanol fraction of the methanol extract of cortex (BF-MEEuC)			BF-MEEuC promoted cell proliferation and differentiation of osteoblast 38% at 1 × 10 ^−1^ mg/ml		
Aqueous fraction of the methanol extract of cortex (AF-MEEuC)			AF-MEEuC promoted cell proliferation and differentiation of osteoblast 26% at 1 × 10 ^−6^ mg/ml		
Geniposidic acid		10 µg/ml in GH inducing test; 1 × 10 ^−1^–1 × 10 ^−11^ mg/ml in MTT test and ALP activity assay; 1 × 10 ^−2^–1 × 10 ^−6^ mg/ml in Praline incorporation test; 1 × 10 ^−1^–1 × 10 ^−7^ mg/ml in inhibition of osteoclast	Geniposidic acid inhibited activities of osteoclast in dose-dependent manner (IC_50_ at 4.43 × 10 ^−7^ M)		
Geniposide			Geniposide promoted cell proliferation and differentiation of osteoblast 35% at 1 × 10 ^−4^ mg/ml. Geniposide promoted synthesis of collagen participating in the matrix formation of osteoblast 20% at 1 × 10 ^− 3^–1 × 10 ^−5^ mg/ml		
Aucubin			Aucubin promoted cell proliferation and differentiation of osteoblast 34% at 1 × 10 ^−4^ mg/ml Aucubin promoted maturation of osteoblast 29% at 1 × 10 ^−11^ mg/ml. Aucubin inhibited activities of osteoclast in dose-dependent manner (inhibited 40% at 1 × 10 ^−3^ mg/ml)		

OVX rats were extensively utilized for investigation of anti-osteoporosis activities of *E. ulmoides*. Reported in 2009, it was found that ethanol extract (100, 300, and 500 mg/kg) of *E. ulmoides* cortex prevents the estrogen deficiency-induced bone loss and deterioration of trabecular microarchitecture, thereby maintaining biomechanical competence of bone in OVX rats [24]. Dietary supplementation of aqueous extract (1.25%, 2.5%, and 5%) of roasted *E. ulmoides* leaf exhibited similar effects in OVX rats [26]. Of note, lignans of *E. ulmoides* have been considered as active fraction groups of anti-osteoporosis activities both *in vivo* and *in vitro* trails. *In vivo*, total lignans (20, 40, or 80 mg/kg) of *E. ulmoides* bark prevented decrease in biomechanical quality of femur, and prevented the deterioration of trabecular microarchitecture, which were in line with above studies. *In vitro*, the lignans treatment (30–500 μg/ml) induced primary osteoblastic cells proliferation and differentiation, and result in inhibition (30, 100, 300 μg/ml) of osteoclastogenesis through an increase in osteoprotegrin (OPG) and a decrease in RANKL expression [26]. This is an early literature that recognizes the anti-osteoporotic effects of *E. ulmoides* in relation to the OPG/RANKL system.

As well as in OVX rats, extracts of the *E. ulmoides* leaves or bark of have been shown to have anti-osteoporotic or bone-enhancing effects in animals in a variety of physiopathological states. Pan et al. in 2014 reported that ethanol extract (300 mg/kg) of the cortex exhibited preventing HLS-induced decrease of bone mass and the deterioration of trabecular microarchitecture, and helping to maintain the structural integrity and biomechanical quality of bone [22]. Another study has also demonstrated that ethanol extract (30 and 100 mg/kg) of the cortex administration into adolescent female rats increased longitudinal bone growth rate and growth plate height, characterized by increasing BMP-2 and IGF-1 expressions in the proliferative and hypertrophic zones [31]. Ethanol extract (495 and 990 mg/kg) of the leaf has also shown anti-osteoporotic effects in senescence-accelerated mice P6 [27]. In lead acetate-induced bone loss rats, ethanol extract (100 mg/kg) of the cortex has been demonstrated *in vivo* trails to be anti-osteoporosis and increased OPG expression and OPG/RANKL ratio, which were in line with the mentioned above *in vitro* study. Nevertheless, there was no significant difference with or without treatment while refer to RANKL mRNA levels [23]. Whether the discrepancy in modulation of RANKL expression attribute to physiopathological context needed to be further investigated. By the way, total glycosides of *E. ulmoides* seed was demonstrated for the first time taken orally (100 mg/kg) increased bone density and bone strength of rat femur in specific‐pathogen‐free (SPF) female rats [32].

### Anti-inflammatory activities of *E. ulmoides*

Last decade has extensively investigated the anti-inflammatory activities of *E. ulmoides* in distinct inflammatory disease models, involving septic inflammation and autoimmune diseases [33–37]. Of note, *E. ulmoides* may be a promising candidate in treating neuroinflammation, as the active components isolated from *E. ulmoides*, such as aucubin, has been profoundly demonstrated alleviating neuroinflammation in Parkinson’s disease, Alzheimer’s disease and seizures *in vivo* [38–40]. Except for the anti-neuroinflammatory activities covered in the neuroprotective section, the rest of the anti-inflammatory activities and its mechanisms were discussed in the following.

Aqueous extract of *E. ulmoides* bark has been extensively investigated for the treatment of lipopolysaccharide (LPS)-stimulation trails *in vitro*. In 2012, it was documented that the aqueous extract of bark treatment (0.05, 0.1 and 0.5 mg/ml) exert inhibition of LPS-induced NF-κB activation, IκB-α degradation, and caspase-1 activation in mice peritoneal macrophages [41]. In LPS-stimulated BV-2 microglial cells, the aqueous extract of bark treatment (2.5, 5, 10, 25, 50 and 100 μg/ml) downregulated the production of pro-inflammatory cytokines, mediators, and reactive oxygen species (ROS). Mechanistic investigation found its effects via modulating MAPKs, PI3K/Akt, and GSK-3β, consequently suppressing NF-κB activation and inducing Nrf2-dependent HO-1 activation [34]. Indeed, oxygen-derived free radicals and high-energy oxidants manifested as mediators of inflammation, and currently antioxidant therapy has already been proposed into treatment of inflammation, which might appreciate the role that *E. ulmoides* plays in alleviating oxidative stress through the Nrf2/HO-1 in favor of anti-inflammation [42]. Lipopolysaccharide is belonging to the pathogen-associated molecular patterns (PAMPs) that can be detected by TLR4 therefore lead to subsequent signalling cause inflammation [43]. Of note, conducted by Koh et al., a critical study established that aqueous extract of *E. ulmoides* cortex exerted anti-inflammation via interfering either of Myd88-dependent (suppress NF-κB, MAPK pathways) or independent pathway (suppress IFN-β and STAT pathway) of TLR-4. The study as well demonstrated the extract, performed with Raw 264.7 cells, suppressed LPS-induced NO production (IC_50_: 356.23 μg/ml) and downregulated expression of iNOS, COX-2, TNF-α, IL-1β and PI3K/Akt/mTOR pathway in a dose-dependent manner [33].

Flavones and polysaccharide are active compound of *E. ulmoides*. In LPS-induced IPEC-J2 cells, flavones (10 μg/ml) from *E. ulmoides* leaf exhibited alleviating LPS induced damages, and mechanistic study unveiled the flavones harbor cytoprotective properties against inflammation approximately via PI3K/NF-κB signaling pathway [44]. In L02 cells transfected with TLR4 overexpression plasmid, polysaccharide (8 μg/ml) of *E. ulmoides* significantly inhibited the increasing expression of TLR4, MyD88, P-p65, and P-IκB-α proteins. The mechanisms referring to the inhibition TLR-4/NF-κB pathway activation by reducing HMGB1 release inhibition [45].

In 2016, first to show effects and mechanism of *E. ulmoides* on rheumatoid arthritis (RA), ethanol extract (dose of raw herbs, 2.7 g/kg) of the cortex in collagen-induced arthritis (CIA) rats reduced the number of Th17-positive cells and downregulated serum IL-17 expression and increased the anti-inflammatory effects of IL-10. In addition, it suppressed the proliferation of synoviocytes and prevented bone tissue damage which characterized by decreased RANKL mRNA whereas increased OPG mRNA expression [37]. The roles of Th17 cells and IL-17 in RA have been well reviewed recently [46, 47]. Afterwards, conducted by the same team, ethanol extract of *E. ulmoides* cortex was fractionated with AcOEt and n-butyl alcohol sequentially, all fractions and extract (each dose of raw herbs at 4 g/kg) increased OPG/RANKL ratio, decreased MMP-9 expression, and inhibited IKK/NF-κB signaling pathway, whereas AcOEt fraction exhibited better improvement effect on OPG/RANKL system than others [35].

An emerging body of research on the anti-inflammatory activities of the active components of *E. ulmoides* has been conducted in the last five years. It was demonstrated that Aucubin suppressed LPS-induced inflammation and apoptosis in cardiac dysfunction mice, and protected against LPS-induced acute pulmonary injury through regulating Nrf2 and AMPK pathways both *in vitro* and *in vivo* [13, 48]. Relatively, geniposide is more extensively utilized to investigate its anti-inflammatory effects and its mechanisms. Geniposide was found inhibiting NLRP3 inflammasome activation via autophagy in BV-2 microglial cells exposed to oxygen-glucose deprivation/reoxygenation [49]. Moreover, geniposide attenuates dextran sulfate sodium-induced colitis in mice via Nrf-2/HO-1/NF-κB pathway [50]. Protocatechuic acid has also been revealed possessing anti-inflammatory activities in LPS-challenged piglets [51]. Although these findings do not directly indicate the anti-inflammatory effects of *E. ulmoides*, they provide a direction for the further investigation of the material basis of the anti-inflammatory activities and mechanisms of *E. ulmoides*.

### Antioxidant activities of *E. ulmoides*

For more than 20 years, the antioxidant activities of *E. ulmoides* has been most extensively studied compared to other pharmacological activities. The leaves, bark and seed of *E. ulmoides* are all medicinal parts with antioxidant activities, and antioxidant assay *in vitro* including DPPH radical-scavenging abilities, ferric reducing antioxidant power and lipid peroxidation inhibition capacity have shown that the leaves are stronger than the bark, while the seeds are the least (Table 2) [52–54]. Total phenolic content and total flavonoid content are considered to correlative with the strength of the antioxidant activity of *E. ulmoides*. Indeed, the total phenolic and total flavonoid content of leaves, bark and seed are in line with the corresponding strength of antioxidant capacity. Other data obtained from tests such as influence of meat color, and metmyoglobin formation in raw pork patties or others comparing the strength of the antioxidant capacity of different medicinal parts of *E. ulmoides* are consistent with the above [54, 55]. Besides, a study reported in 2013 has demonstrated the antioxidant activities of *E. ulmoides* male flowers and identified its main active compound as chlorogenic acid [56]. Of note, Zhang et al. have carried out a comprehensive assessment of the antioxidant capacity of *E. ulmoides* leaves collected monthly during the period of May to October in three years. Determination of active compound content and several antioxidant assays were performed for aqueous extracts of these sample, as well as active compounds therefore obtained a large amount of data. The results showed that August and May were indicated as the best months to harvest contribute to the high antioxidant activities [57].

**Table 2:** Antioxidant activities of *E. ulmoides*

Compounds	Subjects	Chemical composition	Effects	Notes	References
Ethanol extract of *E. ulmoides* leaf (EEEuL)	DPPH radical- cavenging; Fe^2+^-chelating ability; Lipid peroxidation	Total phenolic content: mg CAE/g. Geniposidic acid: 2.14 ± 0.04 mg/g. Epicatechin: 1.93 ± 0.05 mg/g. Chlorogenic acid: 8.45 ± 0.9 mg/g.	EC_50_ of DPPH radical-scavenging: 1.35 ± 0.03 mg/ml	Total phenolic content as chlorogenic acid equivalents (CAE)	[53]
			EC_50_ of Fe^2+^-chelating ability: not detectable		
			EC_50_ of Lipid peroxidation: 16.74 ± 0.09 mg/ml		
Ethanol extract of *E. ulmoides* leaf (EEEuL)	DPPH radical- scavenging; ferric reducing antioxidant power (FRAP); lipid peroxidation inhibition capacity	Total Phenolics Content (TPC): 94.46 ± 17 mg of GAE/g Total Flavonoids Content (TFC): 61.36 ± 0.59 mg of CE/g. Chlorogenic acid: 18.39 ± 0.09 mg/g Caffeic acid: 2.55 ± 0.08 mg/g Protocatechuic acid: 1.60 ± 0.03 mg/g Rutin: 9.99 ± 0.07 mg/g Quercetin: 3.51 ± 0.05 mg/g Kaempferol: 1.10 ± 0.04 mg/g	DPPH scavenging activity: 81.40 ± 3.63% Ferric reducing power EC_1_: 0.72 ± 0.02 mg/ml Lipid peroxidation inhibition capacity: 43.58 ± 3.29%	Total phenolic content as gallic acid (GAE) equivalents Total Flavonoids Content (TFC) as catechin equivalents (CE) equivalents	[54]
Ethanol extract of *E. ulmoides* roasted bark (EEEuRB)		Total Phenolics Content (TPC): 40.07 ± 0.45 mg of GAE/g Total Flavonoids Content (TFC): 16.84 ± 0.20 mg of CE/g. Chlorogenic acid: 7.38 ± 0.12 mg/g Caffeic acid: 1.54 ± 0.05 mg/g Protocatechuic acid: 1.02 ± 0.06 mg/g Rutin: 2.46 ± 0.05 mg/g Quercetin: not detectable Kaempferol: not detectable	DPPH scavenging activity: 16.72 ± 0.25% Ferric reducing power EC_1_: 2.81 ± 0.09 mg/ml Lipid peroxidation inhibition capacity: 26.71 ± 2.21%		
Ethanol extract of *E. ulmoides* seed (EEEuS)		Total Phenolics Content (TPC): 19.11 ± 0.12 mg of GAE/g Total Flavonoids Content (TFC): 7.97 ± 0.11 mg of CE/g. Chlorogenic acid: 0.67 ± 0.01 mg/g Caffeic acid: not detectable. Protocatechuic acid: 0.26 ± 0.03 mg/g Rutin: not detectable. Quercetin: not detectable Kaempferol: not detectable	DPPH scavenging activity: 7.65 ± 0.20% Ferric reducing power EC_1_: 8.43 ± 0.75 mg/ml Lipid peroxidation inhibition capacity: 25.10 ± 1.37%		
Aqueous extract of *E. ulmoides* leaf (AEEuL)	DPPH radical- scavenging	Geniposidic acid Caffeic acid Chlorogenic acid Ferulic acid Quercetin 3-O-sambubioside Rutin Isoquercitrin Ascorbic acid	IC_50_ of AEEuL: 18.9 ± 0.2 μg/ml IC_50_ of Geniposidic acid: > 50 μg/ml IC_50_ of Caffeic acid: 0.8 ± 0.1 μg/ml IC_50_ of Chlorogenic acid: 2.3 ± 0.1 μg/ml IC_50_ of Ferulic acid: 1.6 ± 0.1 μg/ml IC_50_ of Quercetin 3-O- sambubioside: 5.7 ± 0.2 μg/ml IC_50_ of Rutin: 19.8 ± 0.2 μg/ml IC_50_ of Isoquercitrin: 7.2 ± 0.1 μg/ml IC_50_ of Ascorbic acid: 5.9 ± 0.1 μg/ml		[52]

Early in 1998, *E. ulmoides* has been demonstrated dose-dependently (0–0.4 mg/ml) inhibited the enzymatic and non-enzymatic lipid peroxidation of microsomal lipids *in vitro*, and the leaves extract was significantly more potent than the roasted bark extract and raw bark extract in either enzymatic or non-enzymatic peroxidation [58]. Afterwards, these three extracts were assessed on oxidative damage in biomolecules. All extracts (0–1.0 mg/ml) exhibited inhibitory activities in oxidation of deoxyribose in a concentration-dependent manner. In DNA protective trails, only leaf extract (5, 10 μg/μl) exhibited reducing DNA strand-breaking from Fenton reaction. Further in Bleomycin-dependent DNA damage trails (once the extracts reduced the bleomycin- Fe^3+^ to bleomycin- Fe^2+^, DNA damage would occur), the results showed the three extracts of *E. ulmoides* had no significant prooxidant effect while facing bleomycin- Fe^3+^ and the leaf extract could chelate iron ions but had no significant ability to reduce Fe^3+^ to Fe^2+^. These analyses indicated *E. ulmoides* exert antioxidant activities via scavenging hydroxyl radical, function as chelating agent [59].

Over the past two decades, in complement to the above assessment of antioxidant activities, the favorable antioxidant activities of *E. ulmoides* has been demonstrated in biological *in vivo* experiments, with validity against oxidative stress in gastric mucosal injury, chronic hepatotoxicity, diabetes complications, lead-induction, obesity, I/R induced renal and hepatic toxicity etc. [45, 60–65]. Aqueous extract of *E. ulmoides* leaves has been demonstrated been therapeutic on water and VC-deficient diets induced gastric mucosal injury pigs. The extract administration (0.5,1.0 and 2.0 g/kg) suppressed gastric intramucosal thiobarubiturate reactive substances (TBARS) levels, and decreased intramucosal levels of BrdU, 8-OHdG, ssDNA and TUNEL, all of which correlate with oxidative stress-induced DNA adducts and strand breaks [65]. Herein, it is pertinent to mention that there are multiple criteria for evaluating tissue levels of oxidative stress, such as superoxide dismutase (SOD), an antioxidant metalloenzyme that catalyzes the disproportionation of superoxide anion radicals to produce O_2_ and H_2_O_2_; catalase (CAT), its main role is to catalyze the decomposition of H_2_O_2_ into H_2_O and O_2_; malondialdehyde (MDA), a stable metabolite of the free radical-mediated lipid peroxidation cascade; as well as glutathione (GSH) and its functionally related enzymes, an essential antioxidant in the body, it is able to scavenge free radicals in the body. Their relevant functions can be referred to other excellent reviews [66–69]. Indeed, these criteria have been widely utilized in *in vivo* experiments to evaluate the antioxidant activity of *E. ulmoides*. In CCl_4_-induced chronic hepatotoxicity rats, aqueous extract (0.1, 0.5, and 1.0 g/kg) of *E. ulmoides* leaves exhibited increasing GSH content, GSH peroxidase (GPH-Px), GSH reductase (GR) and GSH S-transferase (GST) activities, whereas decreased MDA content in liver. In this context, protocatechuic acid (0.1 g/kg) showed similar effects [61]. In type 2 diabetic mice, aqueous extract (0.187 g/100g of diet) of leaves treatment increased erythrocyte SOD, CAT, GSH-Px activities, whereas it showed no effects on erythrocyte GR activities. The treatment also showed no effects on SOD, GSH-Px, and GR activities in liver and kidney but decreased levels of hydrogen peroxide and lipid peroxide in erythrocytes, liver, and kidney [61].

*E. ulmoides* leaves extracts obtained by other methods have also been investigated. *In vitro*, in rat osteoblastic MC3T3-E1 cells with H_2_O_2_-induced apoptosis, ethanol extract of *E. ulmoides* leaf restrained oxidative damage and increased cell survival rate in a dose-dependent manner, with EC_50_: 25 µg/ml. EC_50_ of downregulating caspases 3, 6, 7, and 9 expression ranging from 12.5 to 25 μg/ml [70]. *In vivo*, performed with piglets, extract (250 mg/kg, eucomman ≥ 20.00%, flavone ≥ 8.00% and chlorogenic acid ≥ 5.00%) of the leaves increased total SOD and GSH-Px in serum and liver. Mechanistic trails unveiled the antioxidant activities approximately be related to the upregulation of Nrf2/TNF-α and the activation of the Nrf2 signalling pathway [71].

The antioxidant activities of various groups of active components of *E. ulmoides* are also well documented. In male fattening lambs, polyphenolic (supplementation with 5 and 10 g/kg of diet) extracted from *E. ulmoides* leaves Increased SOD, GSH-Px in serum and GSH-Px in liver, whereas decreased MDA content in serum and liver [63]. In streptozocin-induced diabetic mice, lignans (20 mg/kg) from *E. ulmoides* leaves upregulated the activity of oxidative stress-related enzymes, involving CAT, GSH-Px, and SOD. Mechanistic investigations revealed the lignans could activate Nrf2/HO-1 signaling to combat against oxidative stress [72]. In diquat-challenged weaned piglets, mechanistic studies unveiled that flavones from *E. ulmoides* interfering through Nrf2 signalling played an important role in regulating oxidative stress in the intestine. The treatment (100 mg/kg) decreased the oxidized glutathione (GSSG) concentration and GSSG/GSH ratio and it increased the protein expressions of nuclear Nrf2 and Keap1, mRNA expression of HO-1, NQO-1, GCLC in the small intestinal mucosa [73]. In blood flow blocking-induced hepatic I/R injury rats, polysaccharide (80, 160 and 320 mg/kg) of *E. ulmoides* decreased ROS, MDA levels and increased SOD levels in liver [45]. In rabbits with I/R induced renal toxicity, the polysaccharides (300 and 600mg/kg) increased SOD, CAT, GSH-Px and GR activities whereas decreased MDA content in the kidney [60]. Collectively, these documents provide direct evidence that the polyphenols, lignans, flavonoids and polysaccharides of *E. ulmoides* are the active components of the antioxidant activities.

As showed in Table 2, numerous active components, involving geniposidic acid, caffeic acid, chlorogenic acid, ferulic acid, quercetin 3-O-sambubioside, rutin, isoquercitrin, ascorbic acid, were capable of antioxidant activities. Their IC_50_ of DPPH radical scavenging capacity were assessed and listed in Table 2. Apart from that, geniposide, aucubin and PG have also been reported alleviating oxidative stress and their antioxidant effects through regulation of Nrf2/HO-1 pathways [38, 74–79]. They were probably the critical compounds in the mechanism of antioxidant potency formation in *E. ulmoides*, but not all of them. In nature, excessive oxidant challenge results in damage to biomolecules. Redox balance is maintained by prevention, interception, and repair, and concomitantly the regulatory potential of molecular thiol-driven master switches such as Nrf2/Keap1 or NF-κB/IκB is utilized for system-wide oxidative stress response [80]. Overall, although few studies have directly shown that the aqueous or ethanol extracts of the leaves or bark of *E. ulmoides* exert antioxidant effects by activating the Nrf2 pathway, studies on both the active fraction groups and the individual active ingredients of *E. ulmoides* have indicated that the Nrf2 pathway is an important target for the antioxidant effects of *E. ulmoides*.

### Neuroprotective activities of *E. ulmoides*

*Eucommia ulmoides *has been established to have protective effect against oxidative stress-induced nerve cell death [81–83]. Both *in vivo* and invitro experiments proved that *E. ulmoides* possessing therapeutic effects on neurodegenerative diseases, involving Parkinson’s disease and Alzheimer's disease [84–86].

Several *in vitro* studies revealed the neuroprotective activities of aqueous extract of *E. ulmoides* bark against oxidative stress. In H_2_O_2_-induced human SH-SY5Y neuroblastoma cells, the aqueous extract (5, 10 and 20 μg/ml), in a dose-dependent manner, inhibited neuronal cell death corresponded to alterations in down-regulation of PARP cleavage and caspase-3 cleavage, up-regulation of Bcl-2 and Bcl-xL activation, as well as the reduction of mitochondrial cytochrome c release. The treatment also attenuated H_2_O_2_-induced phosphorylation of JNK, p38MAPK, ERK1/2, and PI3K/Akt [81]. In 6-hydroxydopamine-induced (6-OHDA) SH-SY5Y cells, aqueous extract of the bark (25, 50 and 100 μg/ml) attenuated oxidative stress through suppressing activation of JNK, PI3K/Akt, GSK-3β, and NF-κB pathways [82]. Lignans from *E. ulmoides* are effective components of neuroprotective activities. In glaucoma rats, the lignans treatment (20 mg/kg) prevented oxidative stress-induced ocular neuropathy role via regulating antioxidant enzymes (CAT, GSH-Px, and SOD) and anti-oxidative stress signaling, including the activation of AMPK and Nrf2-ARE signalling [83].

Alzheimer’s disease, a form of dementia, is a slowly progressing disorder characterized by specific protein accumulations in the brain [87]. Documented in 2009, an *in vitro* study assessed the therapeutic effect of aqueous extract of *E. ulmoides* bark and leaves as well as their components on Alzheimer’s disease. In amyloid-β peptide (Aβ)_25–35_-induced PC-12 cells, both the extract of bark and leaves (each at 0.001–1 mg/ml) antagonized Aβ_25–35_-induced cytotoxicity in dose-dependent manner. Geniposidic acid (0.1–100 μg/ml) and chlorogenic acid (1–100 μg/ml) exhibited significant protective effects on the cytotoxicity of Aβ_25–35_ and seemed to be main active components of *E. ulmoides* bark and leaves against Alzheimer's disease. Phenylpropanoid components from *E. ulmoides*, such as ethyl caffeate and pinoresinol diglucoside, and the flavonoid compounds neither rutin nor quercetin exhibited any cytoprotective effect. Mechanistic studies revealed the neuroprotective mechanisms of *E. ulmoides* extract and its active components referring to inhibiting excessive Ca^2+^ influx, reducing additional lactate dehydrogenase (LDH) leakage and rescuing viability loss [86]. Reported in 2010, a study performed both *in vivo* and *in vitro* has also proved the therapeutic effects of aqueous extract of the bark. In Aβ_25–35_-induced learning and memory impairments mice, the extract (5, 10 and 20 mg/kg) treatment improved the induced learning and memory deficit as well as cognitive impairments. *In vitro*, the IC_50_ of the extract in inhibition of acetyl-cholinesterase (AChE) activity was 172 μg/ml. Ex vivo, inhibition of AChE activity was found effective in the hippocampus and frontal bark [85]. This study indicated the therapeutic effects of aqueous extract of *E. ulmoides* bark via blocking AChE activity. However, there is no more in-depth mechanistic investigation.

Parkinson disease is characterized by death of dopaminergic neurons in the substantia nigra and a pathologic hallmark, termed Lewy body, a neuronal inclusion consisting largely of α-synuclein protein aggregations [88]. In 2015, a study proposed the mechanism of *E. ulmoides* treating Parkinson’s disease might via ameliorating the ubiquitin-proteasome system (UPS). In 1-methyl-4-phenyl-1,2,3,6-tetrahydropyridine (MPTP)-induced Parkinson’s disease mice, ethanol extract (150, 300 and 600 mg/kg) of *E. ulmoides* bark antagonized the loss of striatal neurotransmitters and relieved the associated anomaly in ambulatory locomotor activity induced by MPTP in dose-dependent manner. Further *in vitro* trails, performed with MG132-induced SH-SY5Y cell lines, assessed the neuroprotective activities of the components isolated from *E. ulmoides*, involving botulin, wogonin, oroxylin A, geniposidic and aucubin. The results showed all the components (each at 10 μM) attenuated MPP^+^-induced dysfunction of protease activity and reduced MG132-induced cytotoxicity [84].

In addition to the above, recent years, numerous studies have been conducted to investigate in depth the potency and mechanisms of the neuroprotective effects of the active components of *E. ulmoides*. For instance, aucubin (50 mg/kg) has been reported exerting neuroprotective effects against MPTP-induced Parkinsonian mice in part by reducing inflammation and preserving dopaminergic neurons [40]. Aucubin (50 and 100 mg/kg) has also been revealed inhibiting seizure activity in Pilocarpine (PILO)- induced mice and its action might be related to the reduction of neuroinflammation and the regulation of neurotransmission [39]. Other components of *E. ulmoides* involving geniposidic acid, pinoresinol diglucoside and macranthoin G have well been documented with neuroprotective activities [89–92].

### Hypolipidemic activities of *E. ulmoides*

Sufficient *in vivo* or *in vitro* studies have confirmed the ameliorative effect of the aqueous extract of *E. ulmoides* leaves or the ethanol extract of *E. ulmoides* on hyperlipidemia in non-alcoholic fatty liver disease (NAFLD) or acute liver injury, involving type 2 diabetic, obese and hepatotoxic hyperlipidemia [93–98]. Mechanistic studies found that *E. ulmoides* via down-regulation of mTOR signalling therefore enhancing lysosomal functions and promoting autophagy, result in promotion of liver fat metabolism and hepatoprotection, subsequently exert hypolipidemic effects [95].

Aqueous extract of *E. *ulmoides leaves is widely investigated in hypolipidemic studies. Early in 2006, it has been reported that aqueous extract (0.187 g/100 g diet) of the leaves, in type 2 diabetic mice, significantly lowered the hepatic fatty acid synthase, 3-hydroxy-3-methylglutaryl CoA (HMG-CoA) reductase, acyl CoA: cholesterol acyltransferase (ACAT) activities and furthermore elevated the lipoprotein lipase activity in the skeletal muscle. The plasma and hepatic lipid content were reduced, characterized by lower cholesterol and triglyceride concentrations and the higher plasma high-density lipoprotein cholesterol (HDLC) level [98]. Similar downregulated effects in hepatic fatty acid synthase and HMG-CoA reductase activities as well as the HDLC/total cholesterol ratio were attained while high-fat diet (HFD)-induced hyperlipidemic hamsters were administrated with the aqueous extract of leaves (0.187 g/100 g diet) [97]. Notably, the two studies above indicated aqueous extract of *E. ulmoides* leaves exert hypolipidemic effects via altering lipogenesis, fatty acid β-oxidation and cholesterol metabolism in the liver. Reported in 2010, the hypolipidemic mechanisms of the extract was hypothesized to transforming autonomic nerve activities and causing alteration in thermogenesis and body weight. Indeed, the intraduodenal injection of 1 mg the extract into HFD-induce hyperlipidemic rats elevated epididymal white adipose tissue sympathetic nerve activity (WAT-SNA) and interscapular brown adipose tissue sympathetic nerve activity (BAT-SNA), whereas it decreased gastric vagal nerve activity (GVNA) [93]. Documented in 2019, a study unveiled that the treatment of aqueous extract (100 and 200 mg/kg) of *E. ulmoides* leaves, in HFD-induced lipid dysmetabolism rats, exert hepatoprotective effects against steatosis, accompanied by suppression of ER stress, enhancing lysosomal function and increasing autophagic flux. This promoting autophagy of liver through downregulation of mTOR signalling pathway approximately be the mechanism of hypolipidemic effects of the extract in lipid dysmetabolism. Furthermore, *in vitro* trails performed with palmitate-induce HepG2 cells have established that both aucubin and geniposide (each at 25 μg/mL) harbor hypolipidemic activities and similar with the aqueous extract (50, 100, 250 μg/mL), as the results showed the downregulation of mTOR signalling, alleviation of ER stress and promotion of autophagy [95].

In addition, it has been reported that chlorogenic acid (45.23%)-enriched extract (25 mg/L) of *E. ulmoides* improved the lipid metabolism by the transcriptional activating AMPK and inhibiting downstream targets, such as SREBP2 and HMGCR, to suppress the total cholesterol synthesis and total triacylglycerol levels in HepG2 cells. The IC_50_ of the extract on lipid accumulation is 59.2 mg/L [99]. Of note, the AMPK and TOR pathways are interlinked, opposing signaling pathways involved in sensing availability of nutrients and energy and regulation of cell growth [100]. Accordingly, it is worthwhile to investigate the modulation of two signalling pathways by extract of *E. ulmoides*.

The extract of *E. ulmoides* bark has also been investigated for its hypolipidemic activities. In CCl_4_-induced hepatic lipid accumulation rats, ethanol extract (0.25, 0.5 and 1 g/kg) of *E. ulmoides* bark exerted anti-hyperlipidemic effect via alleviation of ER stress and oxidative stress, which characterized by increase of lysosomal enzymes and hepatic GSH and MDA [94]. Another study, in HFD-induced hepatic dyslipidemia rats, has also proved the hypolipidemic activities of ethanol extract of *E. ulmoides* bark through improving lysosomal activities. Furthermore, invitro trails, performed with palmitate-induced human HepG2 hepatocytes, have shown the extract (100 mg/mL) of the bark inhibited palmitate-induced ER stress, reduced hepatic lipid accumulation [96].

### Other activities

**Fig. 2:**
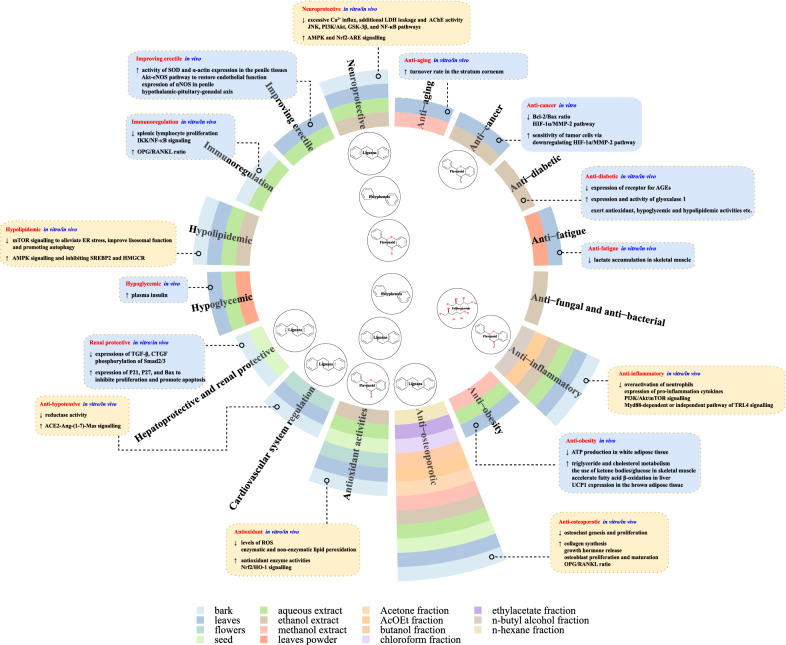
Overview of the proposed modes of action of *E. ulmoides* for its pharmacology activities and research status. On the outside of the ring statistical plot, ↑ indicates upregulation and ↓ indicates downregulation; The ring statistical plot indicates medicinal parts, extraction methods and extraction fraction that *E. ulmoides* have been studied; At the inner side of the ring statistical plot, the compound indicates groups of active ingredients that have been demonstrated having corresponding pharmacological activities

*Eucommia ulmoides *was also reported to possess other pharmacological activities involving anti-obesity, hypoglycemic, anti-diabetic, anti-cancer, immunoregulation, anti-fungal and bacteria, improve erectile, anti-fatigue and anti-aging activities (Fig. 2, involve the pharmacological activities mentioned above). Detailed information was summarized in this section.

#### Anti-obesity activities of *E. ulmoides*

One authoritative definition of obesity is a disproportionate body weight for height with an excessive accumulation of adipose tissue that is usually accompanied by mild, chronic, systemic inflammation [101]. As we have discussed in the section on hypolipidemic activities, extracts of *E. ulmoides* are able to promote the metabolism of lipids such as cholesterol and triglycerides in the liver. Indeed, these functions have the potential to promote the anti-obesity effects of *E. ulmoides*. Besides, there are also some reports that directly demonstrated the capacity of extracts of *E. ulmoides* to reduce the accumulation of body fat and promote the metabolism of substances and energy. Dietary supplementation with aqueous extract (diet containing 3%) of roasted leaves or green leaf powder (diet containing 9%) of *E. ulmoides*, administrated in HFD-induced rats, both minimized increases in body weight and visceral fat in a dose-dependent manner. Both treatments enhanced metabolic function across several organs, including diminishing ATP production in white adipose tissue, accelerating β-oxidation in liver and increasing the use of ketone bodies/glucose in skeletal muscle [102]. Similar results were obtained while treating mice with HFD containing 10% *E. ulmoides* leaves methanol extract. The treatment significantly inhibited body weight, white adipose tissue weight, plasma triglyceride levels and total cholesterol levels. These effects may attribute to asperuloside, which isolated from *E. ulmoides* leaves, as further trails with administration of asperuloside (0.1% dietary supplementation) exhibited analogous effects [103]. The anti-obesity activities and its mechanisms of asperuloside (0.03%, 0.1% and 0.3%) has been compared with roasted *E. ulmoides* leaves aqueous extract (5%). Exclusively, asperuloside administration resulted in increase of non-shivering thermogenesis due to uncoupling protein 1 (UCP1) expression in the brown adipose tissue. Asperuloside may act as a major compound in *E. ulmoides*, relating nearly all metabolic function across several organs, except for the activation of the brown adipose tissue UCP1-induced thermogenesis [104].

#### Hypoglycemic activities of *E. ulmoides*

The hypoglycaemic activities of *E. ulmoides* are seen in diabetic models, whereas *E. ulmoides* has no effect on blood glucose levels in normal physiological states. In streptozotocin-induced diabetic rats, *E. ulmoides* leaves aqueous extract (0.187 g/100g diet) or *E. ulmoides* powder (1 g/100g diet) supplementation treatment showed significant but quite small reduction in plasma glucose, however, occurred with simultaneous the increase in plasma insulin and C-peptide [105]. Another study acquired similar results in lowering blood glucose level as well as enhancing plasma insulin and C-peptide levels, as showed in type 2 diabetic model [98]. Of note, this enhancement of plasma insulin was pathological context dependent. Oral administration of roasted *E. ulmoides* leaves aqueous extract (500 and 1000 mg/kg) notably decreased plasma insulin without affecting blood glucose levels in fructose-drinking rats [106].

#### Anti-diabetic activities of *E. ulmoides*

Diabetes, a chronic degenerative disease, characterized by defective insulin secretion or impairment of its biological actions, or both, causing hyperglycemia and generally results in relatively specific long-term complications affecting the eyes, kidneys, and peripheral and autonomic nervous systems, accounting for more adult cases of vision loss, end-stage kidney disease, and amputations than any other disease [107]. In the section on pharmacological activities above, we have described the therapeutic effects of *E. ulmoides* on complications of diabetic models such as heart disease, oxidative stress, dyslipidemia, and hyperglycemia. Herein, we focus on the modulation of *E. ulmoides* on diabetic advanced glycation end-products (AGEs), and in following another section, the therapeutic effects of *E. ulmoides* on diabetic nephropathy will be discussed.

In streptozotocin-induced diabetic mice, the treatment of ethanol extract (200mg/kg) of *E. ulmoides* bark did not change blood glucose and glycated hemoglobin (HbA1c) levels, whereas there was a significant increase in the protein expression and activity of glyoxalase 1, which detoxifies the AGE precursor, methylglyoxal. The treatment reduced periodic acid-Schiff (PAS)-positive staining, AGEs, methylglyoxal (MGO) accumulation in diabetic mice, and mechanistically, upregulated Nrf2 expression but downregulated expression of receptor for AGE [108].

#### Hepatoprotective and renal protective activities of *E. ulmoides*

In section of hypolipidemic activities, we have discussed the hepatoprotective role of *E. ulmoides* in NAFLD, mainly through downregulation of mTOR signaling thereby improving lysosomal function and promoting autophagy. The protective effects of *E. ulmoides* in acute liver injury have also been described. In the following, we mainly focus on the efficacy of *E. ulmoides* on nephropathy.

Administration of *E. ulmoides* seed ethanol extract (1 g/kg) to streptozotocin-induced type 1-like diabetes in rats, significantly decreased the plasma levels of blood urea nitrogen, creatinine. The treatment markedly attenuated higher expressions of TGF-β levels, connective tissue growth factor (CTGF), and reduced phosphorylation of Smad2/3 in streptozotocin-induced diabetes rats. However, the hyperglycemia-induced overexpression of STAT3 were not reversed [109]. Lignans from *E. ulmoides* bark were considered be highly pharmacologically active compounds, as both *in vivo* and *in vitro* experiments have demonstrated the renal protection against hypertensive renal injury. In SHRs, the lignans treatment (300 mg/kg) significantly decreased N-Acetyl-β-d-glucosaminidase (NAG) enzyme activity, the ratio of albumin to urinary creatinine and the high expression of collagen type III. *In vitro*, the lignans (30 and 90 mg/L) inhibited the proliferation of Ang II-induced renal mesangial cells (RMCs) [9]. Another *in vitro* experiment demonstrated the lignans treatment (20, 40, and 80 mg/L), in ang II-induced RMCs, play a protective role by inhibiting proliferation and promoting apoptosis through the upregulation expression of P21, P27, and Bax [110].

#### Anti-cancer activities of *E. ulmoides*

Several reports have demonstrated the therapeutic effects of *E. ulmoides* on carcinomas. A study showed that the IC_50_ of *E. ulmoides* leaves ethanol extract inhibiting MCF7 cell viability at 48 h and 24 h were about 8.2 and 11.2 mg/mL, respectively, whereas the IC_50_ for purified chlorogenic acid (98.7%) from *E. ulmoides* was only 0.31 mg /mL at 48 h and 0.73 mg /mL at 24 h, much less than that for crude extract [111]. Conducted by Wang et al., the total flavonoid isolated from *E. ulmoides* leaves ethanol extract reduced the viability rate of glioblastomas cells in dose-dependent manner (0–32.0 μg/mL). The IC_50_ and LC_50_ of *E. ulmoides* flavonoid were at 2.76 and 3.15 μg/mL, respectively, showed more effective to inhibit glioblastomas, while comparing to normal human HA cells (IC_50_ at 17.97μg/mL, LC_50_ at 4.71μg/mL). In addition, the flavonoid at 2.0 μg/mL significantly reduced migration and invasion abilities of U251 and U87 in glioblastomas cells. While the flavonoid in combination with radiotherapy, the results showed significantly increase apoptotic of glioblastomas cells, and further investigation found the combination treatment was correlative with the lowest ratio of Bcl-2/Bax, comparing to merely radiotherapy or the flavonoid treatment group. Further analyses indicated an effect of enhancing the sensitivity of glioblastomas cells in radiotherapy by *E. ulmoides* flavonoid, which might refer to downregulating HIF-1α/MMP-2 pathway, as the data showed the expressions of HIF-1α and MMP-2 were further reduced after the combination treatment [112]. In addition, Fujiwara et al. isolated a β-truxinate lignan from *E. ulmoides* leaves, termed, Eucommicin A, which showed excellent inhibitory activities on cancer stem cells (CSCs). Comparing to chlorogenic acid (IC_50_ = 6900 μM), Eucommicin A showed IC_50_ of inhibiting induced CSC-like (iCSCL)-10A viability at 55.0 μM [113].

#### Immunoregulation activities of *E. ulmoides*

A research has been accomplished to explore the modulation of immune system by *E. ulmoides* bark aqueous extract and its active compound genipin. Both the extract (40, 100 and 250 μg/ml) and genipin (10, 20, 50 and 100 μM) exhibited suppressing the proliferation of primary splenic lymphocytes induced by concanavalin A (Con A) or LPS in dose-dependent manner, but not macrophage phagocytosis. *In vivo*, administration of *E. ulmoides* extract (100, 200 and 500 mg/kg) or genipin (10 and 30 mg/kg) into Con A- or LPS-challenged mice showed similar effects, as the splenic lymphocyte proliferation decreased [114]. In addition, *E. ulmoides* has also been shown to have a therapeutic effect on autoimmune diseases such as RA, which has been discussed in the section on anti-inflammatory activities.

#### Improving erectile activities of *E. ulmoides*

Since 2005, it has been reported that *E. ulmoides* aqueous extract (10 and 100 μg/ml) upregulated activity of SOD and α-actin expression in the penile tissues of diabetic rats *in vitro* [115]. And In 2006, conducted *in vivo*, *E. ulmoides* aqueous extract (400 mg/kg) exhibited improving erectile function of diabetic rats via remitting the impairment of myelinated nerve fibers and enhancing the expression of nNOS in penile [116]. Recent years, Fu et al. performed a study to investigate how *E. ulmoides* exert therapeutic effects on erectile Dysfunction. *E. ulmoides* leaves aqueous extract (0.5%, 1% and 2% of HFD) treated into streptozotocin-induced diabetic rats showed attenuating oxidative stress, increasing NO production, activating the Akt-eNOS pathway to restore endothelial function as well as enhancing hypothalamic-pituitary-gonadal axis to improve erectile function [117].

#### Anti-fatigue activities of *E. ulmoides*

The seminal literature reporting on the anti-fatigue activities of *E. ulmoides* may conducted in 1999. It demonstrated that *E. ulmoides* leaves powder supplementation (2.6 g/kg, consist 3% of diet) combination with mechanical training increased the ability to avoid lactate accumulation in skeletal muscle in rats, as characterized by improving 3-hydroxyacetyl-CoA dehydrogenase (HAD) and LDH activities. However, afterwards, this field of study was not explored in-depth [118].

#### Anti-aging activities of *E. ulmoides*

In 1999, a research reported that *E. ulmoides* leaves water soluble methanol extract treatment (mixed into 11 % protein diet at 2.4%), in false aged model rats, led to 20% significant higher turnover rate in the stratum corneum than the control value [119]. Subsequently, one of the iridoid mono-glycosides, namely geniposidic acid, which was an integral part of the extract, while administrated (200 mg/kg) similarly caused a higher turnover rate at 23% in the stratum corneum. Aucubin was an anti-photo-induced aging compound, as it (0.01, 0.1 and 1μg/ml) exhibited decreasing senescence-associated β-galactosidase (SA β-gal) activity in UVB-irradiated human skin fibroblast HS68 cells [120].

#### Anti-fungal and anti-bacterial activities of *E. ulmoides*

Modern studies have demonstrated the anti-fungal and anti-bacterial activities of *E. ulmoides* extracts. Typically, Zhang et al. tested the antifungal and antibacterial effects of *E. ulmoides* ethanol extract at three different concentrations (0.01, 0.10 and 1.00 mg/ml). The results showed *E. ulmoides* extract exhibit high inhibition activities against *Aspergillus fumigatus* fungus, *Acinetobacter *baumannii bacteria, *Pseudomonas aeruginosa* gram-negative and *Staphylococcus aureus* gram-positive bacteria [121].

## Novel components isolated from *E. ulmoides* and their biological activities

**Fig. 3:**
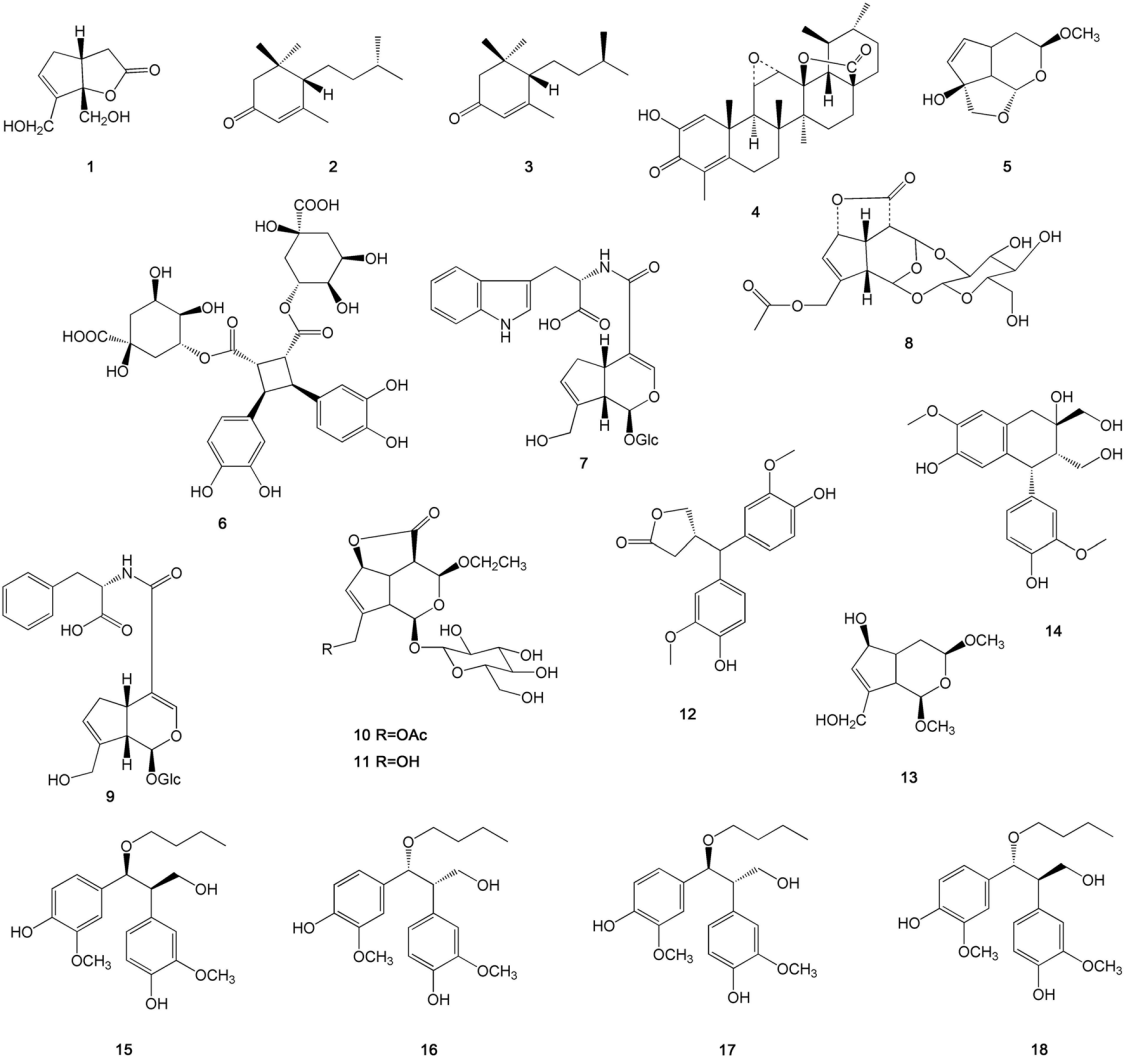
Novel components isolated from *E. ulmoides* in the last twenty years (2000 − 2020)

As a particularly commonly utilized traditional Chinese medicine, *E. ulmoides* has long been one of the most popular drugs investigated. Over the past few decades, phytochemical investigations have demonstrated lignans, iridoids, phenolics, flavonoids, steroid and terpenoids as the main components present in *E. ulmoides* [6]. In the above section, the pharmacological activities of various extracts of the bark, leaves, and flowers of *E. ulmoides* as well as its various groups of active components have been discussed in detail. Herein, the chemical composition of lignans, iridoids, phenolic acids and flavonoids of *E. ulmoides* has been reorganized in Table 3 for reference based on the latest comprehensive review reports of *E. ulmoides* [122]. In addition, this section summarized the novel components that have been isolated from *E. ulmoides* and their biological activities in the last two decades [123–129]. As showed in Figure 3, three monoterpenes (compounds **1**, **2** and **3**), a triterpene (compounds **4**), seven iridoids (compounds **5**, **7**, **8**, **9**, **10**, **11** and **13**), two lignans (compounds **12** and **14**), four phenolics (compounds **15**, **16**, **17**, **18**) and an ester (compound **6**) were isolated from *E. ulmoides*.

**Table 3:** Lignans, iridoids, phenolic acids and flavonoids from *E. ulmoides* bark, leaves, flowers, seed and root

Medicine	Lignans	Iridoids	Phenolic acids	Flavonoids
*E. ulmoides *bark	(+)-medioresinol-di-O-β-d-glucopyranoside Eucommin A Medioresinol (+)-pinoresinol-di-O-β-d-lucopyranoside ( +)-pinoresinol-4'-O-β-d-glucopyranoside Pinoresinol (+)-pinoresinol-4-O-β-d-glucopyranosyl (1 → 6)-β-d-glucopyranoside ( +)-syringaresinol-4'-O-β-d-glucopyranoside Liriodendrin ( +)-syringaresinol 1-hydroxypinoresinol-di-O-β-d-glucopranoside 1-hydroxypinoresinol-4-O-β-d-glucopranoside ( +)-1-hydroxypinoresinol-4'-O-β-d-glucopyranoside 1-hydroxypinoresinol Epipinoresinol (−)-olivil ( -)-olivil-4'-O-β-d-glucopyranoside (−)-olivil-4″-O-β-d-glucopyranoside Olivil-di-O-β-d-glucopyranoside Erythro-guaiacylglycerol-β-coniferylaldehyde ether Threo-guaiacylglycerol-β-coniferylaldehyde ether Citrusin B Dihydrodehydrodiconiferyl alcohol Erythro-dihydroxydehydro-di-coniferyl alcohol Threo-dihydroxydehydro-di-coniferyl alcohol Dehydro-di-coniferyl alcohol 4,γ'-di-O-β-d-glucopyranoside Dehydro-di-coniferylalcohol-γ'-O-β-d-glucopyranoside Hedyotol-C-4',4″-di-O-β-d-glucopyranoside Syringylgylycerol-β-syringaresinolether-4',4″-O-β-d-glucopyranoside Guaiacylgylglycerol-β-syringaresinol ether-4',4″-di-O-β-d-glucopyranoside ( +)-cyclo-olivil Vladinol D 8-hydroxy pinoresinol 9α-hydroxy pinoresinol Hedytol C Hedytol D Pinoresinol vanillic acid ether-di-glucopyranoside Syringaresinol vanillic acid ether-di-gucopyranoside Guaiacylglycerol-8-O-4'-(sinapylaldehyde) ether Guaiacylglycerol-8-O-4'-(sinapylalcohol) ether Guaiacylglycerol-8-O-4'-coniferylaldehyde ether (7S,8S,8'S)-4,9,4',8'-tetrahydroxy-3,3'-dimethoxy-7,9'-monoepoxy lignans	Geniposide Geniposidic acid Genipin Eucommiol I Eucommiol II 1-deoxyeucommiol Epieucommiol Dihydrochalcone Catalpol	Vanillic acid Ascorbic acid Caffeic acid Methyl chlorogenate Chlorogenic acid Coniferol Eucophenoside Erythro-guaiacylglycerol Threo-guaiacylglycerol Catechin Epicatechin Protocatechuic acid methyl ester β-hydroxypropiovanllone C-veratroylglycol 3-hydroxy-4-methoxycinnamaladehyde Catechin-(7,8-b,c)-4α-(3,4-dihyxyphenyl)-2(3H)-pyranone Catechin-(7,8-b,c)-4β-(3,4-dihyxyphenyl)-2(3H)-pyranone 3-hydroxy-1-(3-merhoxy-4-hydroxyphenyl)-propan-1-one Licochalcone A	Quercetin Isoquercitrin (quercetin-3-o-glucoside) Quercetin-3-o-α-l-glucopyranosyl (1 → 2)-β-d-glucopyranoside Quercetin-3-o-xyloseglucoside (quercetin-3-O-sambubioside) Rutin Hyperin (quercetin-3-o-galactoside) Kaempferol-3-o-glucoside (astragalin) Oroxylin Wogonin Wogonside Isoliquiritigenin

Medicine	Lignans	Iridoids	Phenolic acids	Flavonoids
*E. ulmoides* leaves	(+)-pinoresinol-di-O-β-d-glucopyranoside (−)-olivil-4'-O-β-d-glucopyranoside Lariciresinol 8'-methoxy-olivil 8-methoxy-medioresinol 8-hydroxy-medioresinol	Geniposidic acid Aucubin Ulmoidoside Scandoside-10-o-acetate Scandoside methylester Deacetyl asperulosidic acid methyl ester Asperulosidic acid Deacetyl asperuloside Acid Harpagide acetate Reptoside Eucommiol Eucommiol I Eucommiol II 1-deoxyeucommiol Epieucommiol Asperuloside Eucomosides A Eucomosides B Eucomosides C Loganin 7-epi-loganin 8-epi-loganin	Caffeic acid Caffeic acid ethyl ester Ferulic acid P-coumaric acid Methyl chlorogenate Chlorogenic acid 3-o-feruloylquinic acid Catechin Isochlorogenic acid a Isochlorogenic acid c Gallic acid Protocatechuic acid 3-(3,4-dihy-droxyphenyl) propionic acid 3-(3-hydroxyphenyl) propionic acid Pyrogallol 5-methoxy-guaiacylglycerol 5,9-dimethoxy-guaiacylglycerol 9-n-butyl-guaiacylglycerol 9-n-butyl-isoguaiacylglycerol	Isoquercitrin (quercetin-3-O-glucoside) Quercetin-3-O-α-l-glucopyranosyl (1 → 2)-β-d-glucopyranoside Quercetin-3-O-xyloseglucoside (quercetin-3-O-sambubioside) Rutin Hyperin (quercetin-3-O-galactoside) Quercetin 3-O-6″-acetyl-glucopyranoside Kaempferol-3-O-glucoside (astragalin) Kaempferol Kaempherol 3-O-rutinoside (nicotifiorin) Kaempherol 3-O-sambubioside Kaempferol 3-O-6″-acetyl- Glucopyranoside Baicalein Luteolin
*E. ulmoides *flowers		Asperulosidic acid Ethyl ester Daphylloside 4-dihydro-3-methoxypaederoside		Quercetin Isoquercitrin (quercetin-3-O- glucoside) Quercetin-3-O-α-l-glucopyranosyl (1 → 2)-β-d-glucopyranoside rutin Quercetin-3-O-β-d-glucopyranosyl (1 → 2)-β-d-glucopyranoside kaempferol-3-O-glucoside (astragalin) Kaempferol 3-O-6″-acetyl- Glucopyranoside Naringenin Prunin Isorhamnetin-3-O-β-d-glucopyranoside
*E. ulmoides *seed		Geniposidic acid Aucubin Eucommiol II Ulmoidoside A Ulmoidoside B Ulmoidoside C Ulmoidoside D Bartsioside Ulmoidol A	Caffeic acid Chlorogenic acid 3-(3,4-dihy-droxyphenyl) propionic acid Phthalic acid dibutyl ester Phthalic acid bis-(7-ethy-2-hydroxyethyl decyl)-ester Phthalic acid bis-(2-ethy decyl)-ester	
*E. ulmoides* root			Epigallocatechin	Quercetin-3-O-β-d-glucopyranosyl (1 → 2)-β-d-glucopyranoside 4-methyl-7-hydroxycoumarin procyanidin B2 4',7-dihydroxyflavene

Compound **1** was a monoterpene isolated from *E. ulmoides* bark [125]. Compounds **2** and **3** were a pair of megastigmane enantiomers isolated from *E. ulmoides* leaves[128]. Docking-based virtual screening of both compounds showed weak intermolecular interactions with the binding site residues of angiotensin-converting enzyme [130] and angiotensin II type 1 receptor (AT_1_R). Cytotoxic activity was tested for the compound against K562 and HepG2 cells and the result indicated no anti-cancer activity. Compound **4** was a ursane-type nortriterpenoid isolated from *E. ulmoides* leaves and the trails showed no inhibition to proteintyrosine phosphatase-1B (PTP1B) activities [127]. Compounds **8**, **9** and **7** were iridoids isolated from *E. ulmoides* leaves, termed eucomosides A-C [123]. Compounds **5**, **10**, **11**, **13** were iridoids as well but isolated from *E. ulmoides* male flowers and the trails showed no significant promoting proliferation effects on skin fibroblasts cell (ESF-1) [124]. Compounds **12** and **14** were lignans isolated from *E. ulmoides* bark [126]. The neuroprotective activity of both compounds against glutamate-induced HT-22 cells injury was evaluated, and only compound **12** exhibited moderate effect at the concentrations ranging from 10 to 50 μM. Compounds **15**, **16**, **17**, **18** were two pairs of new phenolic enantiomers isolated from *E. ulmoides* leaves[129], but no compounds showed potential cytotoxic activities against Hep G2 *in vitro*. Compound **6** was a quinic acid diester isolated from *E. ulmoides* leaves, termed eucommicin A. The IC_50_ of eucommicin A was 55.0 μM reduced the viability of iCSCL-10A cells [113].

In addition to low-molecular components, there were also several high-molecular components isolated from *E. ulmoides* been reported. In 2016, three novel polysaccharides were isolated and purified from *E. ulmoides*, termed, EUP1, EUP2 and EUP3. Among them, EUP3 has been discovered as the first nonglycosaminoglycan, nonanimal-originated carbohydrate molecule that binds (10 or 100 μg/mL) two pro-angiogenic growth factors, namely, fibroblast growth factor-2 (FGF-2) and platelet-derived growth factor-BB (PDGF-BB), to stimulate angiogenesis [131]. Subsequently, the team designed and fabricated a novel engineered biomaterial, electrospun hydrogel sponge (EGC), based on EUP3 and gelatin to mimic ECM to activate endogenous tissue repair. Animal experiments showed that EGS accelerated the repair of a full-thickness skin wound in mice and induced optimal neo-tissue formation, without the addition of any exogenous GFs, cells or genes [132]. In 2019, the biological applications of EUP3 are being further explored. This study presents a microcarrier system, combining PDGF-BB and PDGF-BB-binding EUP3 for mesenchymal stem cell (MSC) cultivation, which play a role in cell-based therapy strategy towards various diseases and tissue injury [133]. Except for EUP3, EUP1, a novel polysaccharide isolated from *E. ulmoides*, both *in vivo* and *in vitro* trails have demonstrated its anti-inflammatory activities. In LPS-challenged Raw 264.7 cells, EUP1 (10, 25 and 50 μg/ml) reduced the expression of TNF-α in a dose dependent manner. In LPS-induced sepsis mice, EUP1 (10 mg/kg) reserved the upregulation of TNF-α, IL-1β and IL-6 levels in lung tissue [134]. In a study reported in 2004 [135], a novel flavonol glycoside was identified IC_50_ at 2.95 × 10^−7^ M in inhibition test on protein glycation *in vitro*. Besides, four new megastigmane glycosides isolated from *E. ulmoides* leaves, termed eucomegastigsides A-D, were found inhibition ratios of 24.6 ± 0.5%, 29.1 ± 0.6%, 31.2 ± 0.2% and 29.7 ± 0.4% respectively at the concentration of 240 μg/mL against angiotensin converting enzyme [130], which were correlative with the anti-hypertensive activities, and showing moderate activities compared with captopril (98.0 ± 0.1% at 240 μg/mL) [136].

As the second natural rubber resource and one of the main components, *E. ulmoides* rubber, it is worth mentioning that although *E. ulmoides* rubber is not a novel isolated and discovered compound, in the past few years, the great potential application of *E. ulmoides* rubber has received increasing attention in the fields of the environment, agriculture, engineering, and biomedical engineering etc. Very recently, a comprehensive review summarized the novel applications of *E. ulmoides* in diverse fields [137].

## Current developments and limitations of *E. ulmoides*

**Fig. 4:**
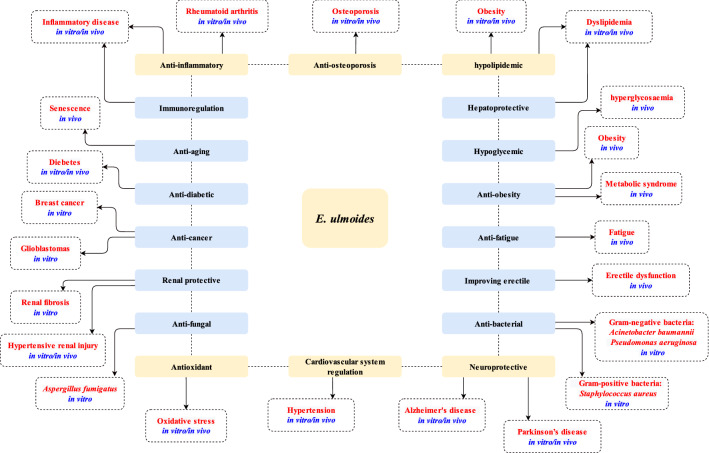
Biological activities of *E. ulmoides* and the potential clinical applications

As described in present review, *E. ulmoides* has been proved to possess a variety of pharmacological activities (Fig. 4). Compared with its recommended therapeutic usages recorded in ancient Chinese medical textbooks, there are still several traditional usages are not estimated by modern pharmacological research, involving intestinal haemorrhoids, vaginal bleeding, dampness and residual draining of urine, abortion, pregnancy bleeding et al. Further investigations are still needed to fully reveal the potential clinical application of *E. ulmoides* and the following aspects are worth addressing.

Firstly, as can be seen in Table 2, there are significant differences in the total phenolics, total flavonoids and other active compounds contents of the seed, leaves and bark of *E. ulmoides*. It is clear that inconsistent chemical composition determines the differences in pharmacological activities. The modern Chinese Pharmacopoeia involving both the bark and the leaves of *E. ulmoides*. However, a variety of studies utilizing the medicinal parts of *E. ulmoides* includes bark, leaves, seed and even male flowers, diversely. Sometimes they are applied as alternative medicines to each other, as they exhibit relatively consistent pharmacological activities and biological mechanisms under certain physiopathological conditions. Overall, both specificities and similarities present in different medicinal parts of *E. ulmoides*. Nevertheless, there is still a lack of systematic investigation unveiling a comprehensive account of the differences in the various components and pharmacological activities of the distinct medicinal parts of *E. ulmoides*.

Secondly, in Wu Yiluo’s “New Compilation of Material Medica”, a decoction of *E. ulmoides* in a 50/50 ratio of wine and water is recorded. The preparation of *E. ulmoides* bark in the Pharmacopoeia is based on reflux extraction with trichloromethane, followed by discarding the trichloromethane solution and refluxing the residue with methanol in water to obtain the extract. In the Pharmacopoeia, the preparation of *E. ulmoides* leaves is done by reflux extraction with 50% methanol. However, for decades, the solvents used for the preparation of extracts from *E. ulmoides* have involved distilled water, methanolic water and ethanolic water. The results obtained from studies of extracts prepared by different processes will not be comparable with each other. In addition, the ultimate efficacy of a drug is influenced by multiple factors other than the preparation process, including planting conditions of soil, geographical location, fertilization methods, harvesting season, etc. For example, it has been shown that harvesting the leaves of *E. ulmoides* in May or August guarantees the best antioxidant activity [57]. In a nutshell. clarification of the chemical profiles and development of standard operating procedures for the *E. ulmoides* products will be crucial in further research.

Thirdly, research on the pharmacological activities of *E. ulmoides*, including anti-cancer, anti-fungal and bacterial, anti-fatigue, anti-ageing, hypoglycaemic and immunomodulatory, is still in its infancy. Studies on the anti-cancer activities of *E. ulmoides* only remain at the stage of *in vitro* trails. Studies on the anti-fungal and anti-bacterial activities of *E. ulmoides* are also limited to *in vitro* trails and lack systematic studies to fully reveal its anti-bacterial and anti-fungal spectrum. *In vivo* tests have demonstrated the anti-fatigue effects of *E. ulmoides*, but the biological mechanism is not yet known. Similarly, while both *in vivo* and *in vitro* tests have demonstrated the anti-ageing effects of *E. ulmoides*, but the mechanism of action needs to be elucidated. It is known that *E. ulmoides* exhibits increasing plasma insulin levels, but this merely results in a small decrease in blood glucose level in diabetic models, and the mechanism of its effect is still unknown. As discussed above, it has been demonstrated that *E. ulmoides* has a lymphoproliferative effect and hence enhances the immune function of the body, however, the mechanism of its effect has not yet been elucidated and its immunomodulatory effect needs to be further explored. In addition, overall, there is a lack of clinical trials on the effectiveness of the various pharmacological activities of *E. ulmoides*.

Fourthly, synergistic effects have been demonstrated between the various compounds inherent in the treatment with *E. ulmoides*, and the interactions may be more complex than thought. For instances, the methanolic extract of *E. ulmoides* has a much stronger effect on collagen synthesis than the acetone fraction of *E. ulmoides* at similar doses. Interestingly, the rest of the n-butanol fraction, the ethyl acetate fraction and the remaining methanol fraction after fractionation were not found to promote collagen synthesis [28]. Thus, there are complex biological mechanisms underlying the *in vivo* conditions of *E. ulmoides* extracts that are not yet known. On the other hand, *in vivo* experiments reported in 2010 showed no hypotensive effect of the iridoids components of *E. ulmoides*, however, several iridoids components isolated from *E. ulmoides* summarized in a 2014 review showed some hypotensive activities in either *in vivo* or *in vitro* experiments [6, 7]. It seemed contradictory, yet it may occur owing to the possible interaction among the multiple components when they are present together *in vivo*, leading to their ineffectiveness. The *in vivo* interaction pattern of multiple components of botanicals remains a major blind spot in this field of research. The synergistic effects *E. ulmoides in vivo* are likewise of great interest.

Fifthly, it is believed that the pharmacological activities of *E. ulmoides* is attributed to diverse chemical components. Indeed, the chemical components isolated from *E. ulmoides* exhibit similar effects to those of *E. ulmoides* extract in numerous pathological contexts. Representatively, a large body of studies have demonstrated the potential biological activities of aucubin involving hypotensive, anti-osteoporotic, antioxidant, hypolipidemic and neuroprotective. In addition, geniposide, geniposidic acid and chlorogenic acid isolated from *E. ulmoides* have also demonstrated multiple pharmacological activities. Although clinical studies are still needed to further establish the effectiveness of these components, it is merit investigation as potential therapeutics.

In addition to the numerous pharmacological studies, *E. ulmoides* had also been widely investigated in other aspects. Above all, iridoids are one of the main active components of *E. ulmoides*. Among them, geniposide and its aglucone genipin by which biological mechanism exerting anti-cancer activities has been discussed in-depth [2, 138–141]. Besides, other pharmacological activities of geniposide have been covered by a large body of excellent reviews. Next, pharmacokinetic investigation, optimization of extraction methods and toxicological trails have also been carried out on *E. ulmoides* [142–147].

## Summary

In summary, extensive *in vitro* and *in vivo* data have revealed that *E. ulmoides* possess multiple excellent biological activities, including cardiovascular system regulation, anti-osteoporotic, anti-inflammatory, antioxidant, hypolipidemic, neuroprotective, hypoglycemic, anti-obesity, anti-diabetic, hepatoprotective and renal protective, anti-cancer, immunoregulation, improving erectile, anti-fatigue, anti-aging, anti-fungal, anti-bacterial, supporting the promising therapeutic application of *E. ulmoides* in various human diseases. For the next decade, more clinical indications would be found with more pharmacological mechanism of *E. ulmoides* being unveiled. We hope this review could provide a scientific basis for further investigations to assess mechanism underlying the effects and clinical applications of *E. ulmoides*.

## Data Availability

Not applicable.

## Abbreviations

8-OHdG: 8-Hydroxy-2 deoxyguanosine
ACAT: CoA: cholesterol acyltransferase
ACE2: Angiotensin-converting enzyme 2
AChE: Acetyl-cholinesterase
AcOEt: Ethyl acetate
AEEuL: Aqueous extract of *E. ulmoides* leaf
AEF-MEEuL: AcOEt fraction of the methanol extract of leaf
AF-MEEuC: Aqueous fraction of the methanol extract of cortex
AF-MEEuL: Acetone fraction of the methanol extract of leaf
AGEs: Advanced glycation end-products
Akt: Protein kinase B
AMPK: Adenosine 5 ‘-monophosphate (AMP)-activated protein kinase
Ang II: Angiotensin II
Ang-(1–7): Angiotensin-(1–7)
AR: Aldose reductase
AT1R: Angiotensin II type 1 receptor
ATP: Adenosine triphosphate
Aβ: Amyloid-β peptide
BAT-SNA: Brown adipose tissue sympathetic nerve activity
Bcl-2: B-cell lymphoma-2
BF-MEEuC: Butanol fraction of the methanol extract of cortex
BMP-2: Bone morphogenetic protein-2
BrdU: 5-Bromo-2-deoxyUridine
cAMP: Cyclic adenosine monophosphate
CAT: Catalase
CIA: Collagen-induced arthritis
Con A: Concanavalin A
COX-2: Cyclooxygenase-2
CSCs: Cancer stem cells
CTGF: Connective tissue growth factor
DNA: DeoxyriboNucleic acid
DPPH: 1,1-Diphenyl-2-picrylhydrazyl
*E. ulmoides*: *Eucommia ulmoides* Oliv.
EC50: Concentration for 50% of maximal effect
EEEuL: Ethanol extract of *E. ulmoides* leaf
EEEuRB: Ethanol extract of *E. ulmoides* roasted bark
EEEuS: Ethanol extract of *E. ulmoides* seed
EF-MEE: Ethylacetate fraction of the methanol extract of cortex
eNOS: Endothelial nitric oxide synthase
ERK1/2: Extracellular signal-regulated kinase 1/2
GH: Growth hormone
GPH-Px: GSH peroxidase
GR: GSH reductase
GRF: Growth hormone releasing factors
GSH: Glutathione
GSK-3β: Glycogen synthase kinase-3β
GST: GSH S-transferase
GVNA: Gastric vagal nerve activity
HAD: 3-Hydroxyacetyl-CoA dehydrogenase
HbA1c: Glycated hemoglobin
HDLC: High-density lipoprotein cholesterol
HFD: High-fat diet
HLS: Hind limb suspension
HMGB1: High mobility group protein
HMG-CoA: 3-Hydroxy-3-methylglutaryl CoA
HO-1: Heme oxygenase-1
I/R: Ischemia/reperfusion
IC50: 50% Inhibiting concentration
iCSCL: Induced CSC-like
IFN: Interferon
IGF-1: Insulin-like growth factors-1
IL: Interleukin
iNOS: Inducible nitric oxide synthase
IκB: Inhibitor of NF-κB
JNK: C-JunN-terminalkinase
LDH: Lactate dehydrogenase
LPS: Lipopolysaccharide
MAPK: Mitogen-activated protein kinase
MDA: Malondialdehyde
MEEuC: Methanol extract of *E. ulmoides* cortex
MEEuL: Methanol extract of *E. ulmoides* leaf
MF-MEEuL: Methonal fraction of the methanol extract of leaf
MGO: Methylglyoxal
MMP-2: Matrix Metallopeptidase 2
MMP-9: Matrix metallopeptidase 9
MPTP: 1-Methyl-4-phenyl-1,2,3,6-tetrahydropyridine
mTOR: Mammalian target of rapamycin
Myd88: Myeloiddifferentiationfactor 88
NAFLD: Non-alcoholic fatty liver disease
NAG: N-Acetyl-β-d-glucosaminidase
NF-MEEuL: N-hexane fraction of the methanol extract of leaf
NF-κB: Nuclear factor kappa-B
NLRP3: Nod-like receptor protein 3
nNOS: Neuronal nitric oxide synthase
NO: Nitric oxide
Nrf2: Nuclear factor E2-related factor 2
OHDA: 6-Hydroxydopamine-induced
OPG: Osteoprotegerin
OVX: Ovariectomy
PAMPs: Pathogen-associated molecular patterns
PARP: Poly-ADP-ribosepolymerase
PAS: Periodic acid-Schiff
PG: ( +)-Pinoresinol di-β-d-glucoside
PI3K: Phosphatidylinositol 3-kinase
PILO: Pilocarpine
P-IκB: Phospho-IκB
P-p65: Phospho-p65
PTP1B: Proteintyrosine phosphatase-1B
RA: Rheumatoid arthritis
RANKL: Receptor activator of nuclear factor-κB ligand
RMCs: Renal mesangial cells
ROS: Reactive oxygen species
SA β-gal: Senescence-associated β-galactosidase
SHRs: Spontaneously hypertensive rats
Sirt1: Sirtuin
SMC: Smooth muscle cell
SOD: Superoxide dismutase
SPF: Specific‐pathogen‐free
ssDNA: Single-stranded DNA
STAT: Signal transducer and activator of transcription
TBARS: Thiobarubiturate reactive substances
TGF-β: Transforming growth factor-β
Th17: T helper cell 17
TLR4: Toll-like receptors 4
TNF-α: Tumor necrosis factor-α
TUNEL: TdT-mediated dUTP-biotin nick end labeling
UCP1: Uncoupling protein 1
UPS: Ubiquitin–proteasome system
VC: Vitamin C
WAT-SNA: White adipose tissue sympathetic nerve activity
β3-AR: β3-Adrenergic receptor
